# A dataset of mentorship in bioscience with semantic and demographic estimations

**DOI:** 10.1038/s41597-022-01578-x

**Published:** 2022-08-02

**Authors:** Qing Ke, Lizhen Liang, Ying Ding, Stephen V. David, Daniel E. Acuna

**Affiliations:** 1 School of Data Science, City University of Hong Kong, Kowloon, Hong Kong; 2 School of Information Studies, Syracuse University, Syracuse, New York 13244 USA; 3 School of Information, University of Texas at Austin, Austin, Texas 78712 USA; 4 Oregon Hearing Research Center, Oregon Health and Science University, Portland, Oregon 97239 USA

**Keywords:** Careers, Authorship

## Abstract

Mentorship in science is crucial for topic choice, career decisions, and the success of mentees and mentors. Typically, researchers who study mentorship use article co-authorship and doctoral dissertation datasets. However, available datasets of this type focus on narrow selections of fields and miss out on early career and non-publication-related interactions. Here, we describe Mentorship, a crowdsourced dataset of 743176 mentorship relationships among 738989 scientists primarily in biosciences that avoids these shortcomings. Our dataset enriches the Academic Family Tree project by adding publication data from the Microsoft Academic Graph and “semantic” representations of research using deep learning content analysis. Because gender and race have become critical dimensions when analyzing mentorship and disparities in science, we also provide estimations of these factors. We perform extensive validations of the profile–publication matching, semantic content, and demographic inferences, which mostly cover neuroscience and biomedical sciences. We anticipate this dataset will spur the study of mentorship in science and deepen our understanding of its role in scientists’ career outcomes.

## Abstract (summary)

**Table:** 

Measurement(s)	Semantics • Gender • Race
Technology Type(s)	unsupervised machine learning • supervised machine learning
Factor Type(s)	FirstName • FullName • MAGPaperID • DOI • PMID • TFIDFVector • SpecterVector • PID • NeighborPID • SpecterDistance • MiddleName • LastName • Institution • InstitutionMAGID • ResearchArea • ORCID • MAGAuthorID • MenteeID • MentorID • MentorshipType • StartYear • StopYear • gender • race • CID

## Background & Summary

Mentorship is a form of guidance provided by a more experienced person (mentor) to a less seasoned one (mentee). Likewise, mentors in science draw from their experiences to help mentees–who often are early-career researchers–navigate various issues inside and outside of academia. Mentorship is a crucial phase in a scientist’s development that has long-term effects throughout her career. Mentorship can occur formally through doctoral and postdoctoral advisor–advisee relationships or informally through collaborations. Mentees not only learn new knowledge and skills from mentors but also get involved in mentors’ social connections^1^. Numerous studies have pointed out the association between mentor’s characteristics and mentee’s academic success, like productivity^2–4^, career preference and placement^2,5,6^, mentorship fecundity^7,8^, and impact^9^. Despite the large role of mentorship and interest in studying it, previous studies have relied on single-field datasets and indirect signals of mentorship (e.g., co-authorship) and therefore have limited generalizability. Large, curated, and open datasets on mentorship have the potential of bringing significant benefit to our understanding of the phenomenon, similar to how citation and publication datasets have accelerated the emerging field of science of science^10,11^.

Studying mentorship requires access to a broad set of relationship types, including publication. There are a few data sources for mentorship in science (Table 1); here, we list a handful of them. The Mathematics Genealogy Project (MGP)^12^ is an online database for academic genealogy only in mathematics, though more broadly construed to include “mathematics education, statistics, computer science, or operations research”. MGP lacks publication records. The Astronomy Genealogy Project is a similar online database confined to astronomy that also does not have publication information^13,14^. ProQuest is a database of theses and dissertations predominantly from the US^15^. Although it is multi-disciplinary, it does not disambiguate researchers, making it hard to link advisor and advisee and construct lineages. Also, it does not provide publication information. More importantly, ProQuest is not publicly available, and its access is rate-limited. Apart from genealogy and thesis data, other researchers have proposed to use paper co-authorships as indirect signals of mentorship^16^. However, mentorship can start much earlier than publishing works, and it does not necessarily lead to publications^17^. To summarize, datasets about mentorship in science are in general fragmented.

**Table 1:** Comparison of existing datasets of mentorship in science with ours (Mentorship).

Database	Discipline	Country	Tree	Publication data	Open	Demographics	Semantics
Mentorship	all	world-wide	✓	✓	✓	✓	✓
Academic Family Tree	all	world-wide	✓	✓	✓	✗	✗
Mathematics Genealogy Project	Math	world-wide	✓	✗	✓	✗	✗
Astronomy Genealogy Project	Astronomy	world-wide	✓	✗	✓	✗	✗
ProQuest	all	US	✗	✗	✗	✗	✗

Here, we start from the Academic Family Tree (AFT) website^18^ and extend it to create a large-scale dataset of mentorship relationships in science. The AFT is an online portal for mentorship in science. We match each AFT profile to the Microsoft Academic Graph (MAG) we retrieved in September 2020, a leading bibliographic database^19^. Moreover, we apply natural language processing techniques to extract semantic representations of researchers based on deep learning content analysis of their publications. Given the recent interest to understand the role of gender and race/ethnicity in science^20^, we also provide estimations of researchers’ demographics. Compared to existing databases, our dataset, Mentorship (Mentorship with Semantic, Hierarchical, and demographIc Patterns), covers a wide range of disciplines with a richer set of features, making it ideal for studying generalizable mentorship patterns. We expect it to be the base of future studies covering various aspects of scientific mentorship, including semantic and demographic factors.

## Methods

### Data sources

The AFT website displays researchers’ profile information, like direct academic parents and children and a limited set of publication records in the PubMed. Originally focused on neuroscience^21^, AFT has been expanding to other areas such as chemistry, engineering, and education. As a crowd-sourcing website, contents on AFT are contributed by registered users. Contributions can be diverse, from adding a new researcher to adding mentors, trainees and collaborators of an existing researcher. Visitors can also indicate whether the website has correctly matched a profile with a publication. Due to the crowd-sourcing nature, researchers on AFT may not be a representative sample of the academic population.

In AFT, the user-contributed data are stored in a database consisting of several tables that are available online^22^. These tables are the starting point for the present work. In particular, we use four tables: (1) the people table storing researchers’ basic information, including person’s ID, name, degree, research area, etc.; (2) the connect table detailing mentorship relationships, including its ID, mentee and mentor person IDs, mentorship type (e.g., PhD, postdoctoral advising), and when and where the mentorship occurred; (3) the authorPub table enumerating researchers and their papers as well as meta data of papers; and (4) the locations table listing institutions and their geolocations.

We use the MAG dataset to find papers of AFT researchers. MAG contains information about papers, authors, journals, conferences, affiliations, and citations. One advantage of MAG is that all entities have been disambiguated and associated with identifiers. This dataset has been used in several recent works for author- and venue-level analyses^20,23^. Here we use a version of the MAG obtained in September 2020, which contains 183214248 journal articles, conference papers, and documents with type unknown. These documents in total have 509686489 authorships, among which 243010210 (47.7%) have affiliations. At the author level, there are 193991023 unique authors, and 66729887 (34.4%) of them have at least one paper with affiliation. Four tables in MAG are used: (1) the Affiliations table that lists institution related information; (2) the PaperAuthorAffiliations table that records the name and the affiliation of each authorship; (3) the Authors table that contains author information including names; and (4) the Papers table that consists of paper-related metadata such as digital object identifier (DOI).

**Fig. 1:**
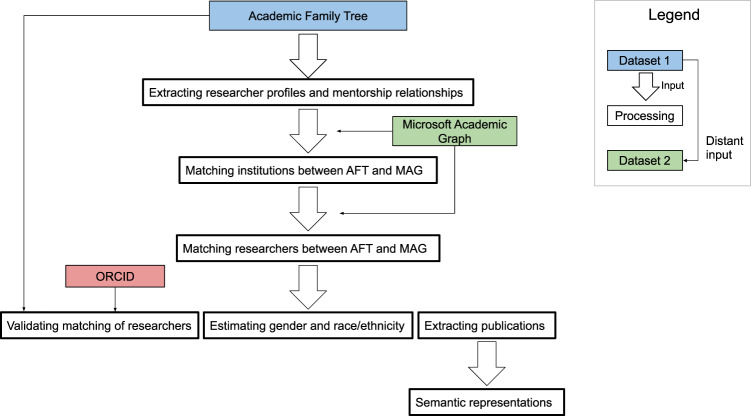
Flowchart of the dataset generation process.

Figure 1 provides an overview of how these data sources are used to assemble the dataset presented in our work.

### Normalizing researcher profiles

The people table contains 778367 researchers, uniquely identified by person IDs. We clean this table by ignoring (1) researchers without a first name or last name; (2) researchers who have the same name, institution, and major research area but different IDs as they are likely duplicates; and (3) researchers whose first, middle, or last name contain characters that are not likely to appear in a name, such as “&” and “;”. These steps leave us with 774733 (99.5%) researchers.

Besides person IDs that are used internally in AFT, there are about 1600 researchers whose Open Researcher and Contributor ID (ORCID), a persistent identifier to uniquely identify authors^24^ with the focus in biosciences, are available. Although this is a small fraction (0.2%), we use this information for later validation of our methods. This ORCID information needs cleaning before using it as it contains various “orcid.org” prefixes (“https://orcid.org/”, “http://orcid.org/”, and “orcid.org/”) and wrong format, which are manually corrected.

### Extracting mentor-mentee pairs

From the connect table, we filter out mentorship pairs where mentee’s person ID or mentor’s person ID are not present in the curated list of researchers generated in the previous section. We then drop duplicate records and ignore records where the same relationship ID corresponds to a different mentee or mentor’s ID. We obtain 743176 mentorship pairs among 738989 researchers.

### Matching institutions between AFT and MAG

To facilitate matching AFT researchers with MAG authors, we first match institutions. To do so, we generate a list of rules to normalize AFT institution names iteratively. More specifically, we perform a greedy matching where we sequentially select the unmatched AFT institution with the largest number of researchers associated with it. We then apply several rules to normalize the name so that we can find it in the MAG institution list (see Table 2 for the rules). For institutions that cannot be matched using these rules, we manually search them in the MAG if they have at least 200 researchers and discard the remaining institutions. These steps are iterated until no more matches are possible.

**Table 2:** A list of rules to normalize AFT institution names used to match with MAG institutions.

replace “,” with “-”
replace “,” with “AT” then replace “-” with “·”
replace “&” with “&”
replace “,” with “·”
remove “THE” then replace “-” with “-”
replace “AT” with “·”
replace “AND” with “&”
extract text in parentheses then replace “UNIVERSIDAD DE” (“UNIVERSIDADE DE”) with “UNIVERSITY OF”
remove accents
text before “,”
replace “-” with “-”
replace “,” with “·”
replace “&” with “AND”
replace “-” with “·”
replace “AND” with “·”
replace “IIT” with “INDIAN INSTITUTE OF TECHNOLOGY”

### Linking AFT researchers to MAG authors

As described before, one unique feature of our dataset is that we provide lists of publications authored by AFT researchers. One motivation behind this is to access the entire co-authorship network of researchers and potentially understand the topics, venues, and citation dynamics of this network. While AFT already has publication information, it is limited to PubMed only. By matching to MAG, we can access all research areas that are not limited to biomedicine.

There are two main strategies we follow to find matches. One approach is to find, for each mentor-mentee pair, the list of MAG papers where both of their names appear as co-authors. The other strategy is to match AFT researchers using their names and affiliation information. This second strategy is necessary because some mentees have not published a paper with a mentor yet.

We first elaborate on the first strategy: matching by co-authorship. This strategy involves the following three steps:

1. First, we prepare a list of mentor-mentee name pairs. To do so, for each AFT researcher, we consider her full name as presented in the AFT. If the first name has more than one character (i.e., not first initial), we also consider two possible variations: (1) first name, middle initial, last name; and (2) first name and last name. For a mentor-mentee pair, we then enumerate all possible name pairs.
2. Second, we scan the MAG to collect papers where the name pair of two co-authors appear in the list of name pairs prepared in the first step. Specifically, for a MAG paper, we collect its co-author names from the PaperAuthorAffiliations and Authors tables. Then, we use the nameparser Python library^25^ to parse a full name into first, middle, and last name. (Author names in the MAG are given as single text.) Next, we consider all possible name pairs of two co-authors and check if each pair is presented in the list of AFT name pairs prepared in the first step. Note that we only consider conference papers, journal articles, and unknown when performing the matching, ignoring the other five types of documents presented in MAG: book chapter, book, dataset, patent, and repository.
3. After scanning the MAG, we obtain a list of associated papers and the MAG author IDs for the mentor and the mentee for each mentor-mentee pair. In total, 359238 AFT researchers have MAG papers associated with them and have at least one corresponding MAG author ID. Among these researchers, 295630 (82.3%) have only one MAG author ID. For the rest, although multiple MAG ids are associated with them, only one of the ids accounts for more than half of the published works for the vast majority of those researchers. Therefore, we assign the most common MAG author ID to an AFT researcher if there is a single majority (98% of cases). We drop the remaining 2% and result in a total of 353377 AFT researchers linked to MAG using co-authorship-based matching.

Next, we match the remaining 421356 unmatched researchers with MAG using their name and institution information. The procedure is similar to co-authorship-based matching. First, we collect, for an AFT researcher, all possible name-institution pairs, by considering her name variations and institutions presented in the profile and mentorship tables (Fig. 1). We then aggregate those pairs across all researchers. Note that for only 928 (0.2%) unmatched researchers, their name-institution pairs are not unique. Next, we scan the MAG to find papers where the co-authors’ name-institution pairs are in the prepared list of name-institution pairs. Through this way, we additionally match 141078 researchers, with the total matched researchers reaching to 494455 (63.8%).

### Estimating semantic representations

Our efforts so far have yielded a list of papers for each AFT researcher who we can match in MAG. Next, we use the titles and abstracts of these papers to construct vector representations of the researcher. Such models can capture semantics, allowing us to apply them in a wide range of scenarios such as comparing the *content* between researchers^8^, recommendation^26^, and matchmaking of scientists^27^. Here we provide two types of representations; one is based on standard term frequency-inverse document frequency (TF-IDF) vectors, and the other is based on modern deep learning embeddings.

#### TF-IDF representation

The subset of researchers who we can match in MAG published a total of 16942415 papers in MAG. We concatenate the titles and abstracts of these papers. Then using scikit-learn^28^, we preprocess the concatenated text by removing English stop words as well as words appearing only once and apply the TF-IDF transformation. This preprocessing results in a 16942415 × 2275293 sparse matrix, with each row corresponding to a paper and each column a term. The vector of a researcher is the centroid (average) of the TF-IDF vectors of her documents.

#### Deep learning embedding

We employ SPECTER^29^, a representation learning algorithm for scientific documents, to obtain dense vector representations of papers. We concatenate titles and abstracts and use the implementation reported in^30^. Each article is represented by a dense vector of 768 dimensions, resulting in a dense 16942415 × 768 matrix for all documents. The vector of a researcher, again, is the average of the vectors of her papers.

### Estimating gender and race/ethnicity

Gender in science has become an important subject of study^20^. Here we provide researchers’ gender information inferred from their first names. To do so, we encode the character sequence using both the full string and sub-word tokenization as created by a pre-trained BERT model^31,32^. The output of the BERT model is passed through a pooling layer which creates a vector of 768 elements. This vector is then passed through a dropout layer and softmax layer to produce the final gender predictions. We have three genders in our dataset, two legal labels (female and male) and one unknown label, which attempts to capture potentially non-binary genders. For the training data, we use a combination of datasets. One dataset provides predicted gender of author names in the Author-ity 2009 dataset using the Genni and SexMac tools^33^. We only maintain data points where Genni and SexMac agree with each other. This filtering step left us with 2793982 labeled data points. Another dataset for training comes from the Social Security Administration (SSA) and is about popular newborn names and their gender^34^. The SSA dataset contained 95026 names labeled as “male” and “female”. To reduce the generalization error, we sample each class from the aggregated dataset and obtain a relatively balanced dataset with 1500000 data points (male: 600000, female: 600000, unknown: 300000). When training, we sample each of all three labels equally. We use 80% for training and 20% for validating. The classes in both splits are also balanced.

We also provide race/ethnicity information of researchers inferred from their full name using a similar architecture. The deep learning architecture is identical to the one used in the gender prediction above: BERT → Max Pooling → Dropout → Softmax. We combine two data sources as our training set. The first one contains the predicted ethnicity of authors in the Author-ity 2009 dataset using the Ethnea tool^35^. We map the predicted categories into four groups: Asian, Hispanic, Black, and White using the mapping described in Table 3. The second dataset consists of name and ethnicity information extracted from personal profiles on Wikipedia^36^. We map the Wikipedia labels into the same four categories of ethnicity listed before. Finally, we get a dataset with 720000 data points (black: 180000, Asian: 180000, Hispanic: 180000, white: 180000). The training and validation schedule is similar to the one followed for the gender prediction.

**Table 3:** Mapping between race categories in the Ethnea and ours used for prediction.

Race for prediction	Race in Ethnea
Asian	Arab, Chinese, Indian, Indonesian, Israeli, Japanese, Korean, Mongolian, Polynesian, Thai, Vietnamese
White	Baltic, Dutch, English, French, Greek, German, Hungarian, Italian, Nordic, Romanian, Slav, Turkish
Hispanic	Caribbean
Black	African

Both models are incorporated in our Python package demographicx^37^.

## Data Records

**Fig. 2:**
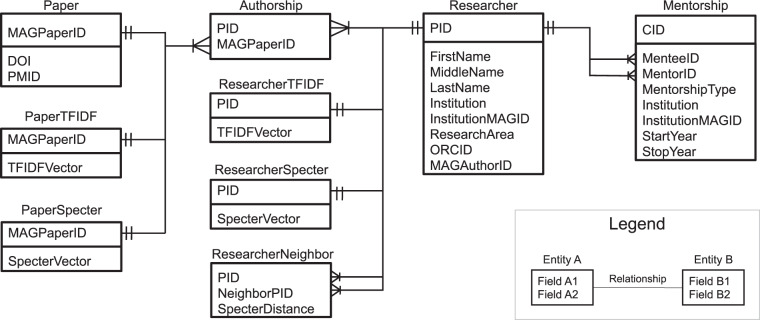
Entity-relationship diagram of our dataset.

The resulting dataset^38^ has 10 main tables, shared as the files described below. Figure 2 presents the entity-relationship diagram of these tables.

1. researcher.csv is a comma-separated values (CSV) file listing 774733 researchers and contains the following variables: person ID (PID), first name, middle name, last name, institution, institution MAG ID, research area, ORCID, and MAG author ID. We also provide an auxiliary file named first_name_gender.csv that maps first name to inferred gender and an auxiliary file called full_name_race.csv that maps full name to inferred race/ethnicity.
2. mentorship.csv contains mentorship relationships between researchers and has 8 variables: relationship ID (CID), mentee’s person ID, mentor’s person ID, mentorship type, the institution where the mentorship happened, institution MAG ID, and the start year and stop year of the interaction.
3. authorship.csv lists all the MAG paper IDs of each researcher and has two columns: person ID (PID) and MAG paper ID.
4. paper.csv lists 3 types of IDs of each paper: MAG ID, PubMed ID (PMID), and DOI.
5. paper_tfidf.npz stores the sparse matrix for paper TF-IDF vectors in Compressed Sparse Row format.
6. researcher_tfidf.npz stores the sparse matrix for researcher TF-IDF vectors in Compressed Sparse Row format.
7. paper_specter.pkl stores SPECTER vectors of papers in the Pickle format.
8. researcher_specter.pkl stores SPECTER vectors of researchers.
9. researcher_neighbor_specter.csv lists the 9 nearest researchers and the distances to them of each researcher based on SPECTER vectors. It has 3 columns: person ID (PID), the neighbor’s person ID (NeighborPID), and their distance (SpecterDistance).
10. coauthored_papers.csv contains all the MAG papers where mentees and mentors are coauthors. The columns are relationship ID (CID), mentee and mentor’s ID, MAG paper ID, mentee and mentor’s MAG author IDs as presented in the paper.

**Fig. 3:**
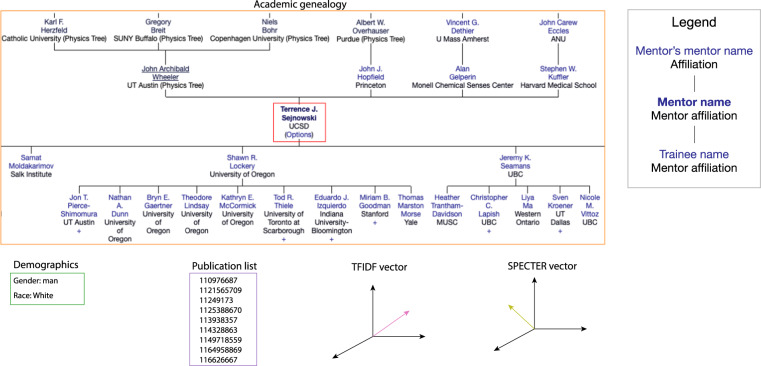
Different types of data available for an exemplar researcher (Terrence J. Sejnowski).

Figure 3 provides a researcher-centric view of the different types of data available in our dataset.

## Technical Validation

### Validation of gender and ethnicity estimation

We report in Table 4 the performances of our gender prediction algorithm on the validation set and the SSA set. To validate the “unknown” class, we used “unknown” labels from Authori-ty for names in the SSA dataset labeled “unknown” in the Authori-ty dataset. For both sets, our algorithm has good performances for all three categories. Applying the algorithm to our dataset, Table 5 presents the numbers of researchers by gender.

**Table 4:** Performances of our gender prediction.

	Validation set			SSA names		
	F-1	accuracy	AUROC	F-1	accuracy	AUROC
Male	0.961	0.972	0.993	0.813	0.771	0.954
Female	0.975	0.979	0.996	0.915	0.885	0.965
Unknown	0.889	0.862	0.966	0.504	0.664	0.860

**Table 5:** Number of researchers by gender. Here, the gender of the researcher is estimated by an algorithm using their first name.

Gender	# researchers
male	374199
female	264263
unk	135732

We acknowledge that there could be a great deal of noise and bias in this estimation. However, we believe it is better to open our algorithm to the community instead of analyzing proprietary software that does not publicize data used and performance metrics.

Similarly, we test our race/ethnicity prediction algorithm on the validation set and the Wikipedia dataset, obtaining good performances for all four groups (Table 6). Table 7 presents the number of researchers by predicted race/ethnicity using our algorithm.

**Table 6:** Performances of our race/ethnicity prediction.

	Validation set			Wikipedia		
	F-1	accuracy	AUROC	F-1	accuracy	AUROC
Black	0.976	0.999	0.999	0.987	0.999	0.996
Hispanic	0.936	0.928	0.990	0.822	0.788	0.964
White	0.907	0.902	0.983	0.850	0.856	0.963
Asian	0.941	0.931	0.989	0.859	0.843	0.962

**Table 7:** Number of researchers by estimated race/ethnicity.

Race/ethnicity	# researchers
White	508923
Asian	177649
Hispanic	68664
Black	18958

To further validate the performance of both model, we gather names with both gender and ethnicity label from the validation dataset. We breakdown the names into subgroups including black male, black female, Hispanic male, Hispanic female, white male, white female, Asian male, and Asian female. We tested both the gender and ethnicity prediction algorithm on the new validation dataset and calculate F-1 and precision for each category. The performance for each subgroup is shown in Tables 11, 12.

Even though the model has achieved great performance, we found that African American names are under-represented in the training data set. Since the majority of black names are from outside the U.S., the model made predictions largely based on information about African names outside of the U.S. and might suffer from poor performance when predicting African-American names. Due to the sensitive nature of names and ethnicity, it is hard to find full names of African American names. However, we retrieved 340 names from the Black In Neuro website^39^ to estimate the extend of the issue. The average probability of predicting a name to be black was 19.5%, with many names being classified as white names. While names retrieved from Black In Neuro are small and might introduce selection bias, the validation suggests that the ethnicity predictions are poor for African-American names. To improve upon this performance, we created a second model that uses only the surnames reported on the U.S. Census^40^. The performance of this second model was significantly better on the Black in Neuro dataset (30%). The validation on the U.S. Census reveals that this model has worse performance that the first model above (validation data: Black F1: 0.53, Asian F1: 0.64, Hispanic F1: 0.692, White F1: 0.52). We leave it to the user to determine which of the two models better serves their analysis.

Finally, we compared our estimations and our software package against other two popular solutions used in our articles. The first such solution is the genderize.io API. This tool is paid if more than 1,000 queries need to be performed per day. Only a sample of 2,000 random names, our tool is on par with the performance of genderize.io: contrast Tables 4, 9. Similarly, we test our race estimation against a popular Python package called ethnicolr with the North Carolina Voter Registration Data, which includes labels for both gender and ethnicity. Our method and tool are 0.01 to 0.67 raw F-1 score above in performance: contrast Tables 10, 11. Importantly, our tool, demographicx, is openly available and tested for any body to try.

### Validation of mentorship

Our dataset covers mentorship relationships in multiple disciplines. Table 8 presents the top 20 most represented areas. Neuroscience is the one with the largest number of researchers, given that AFT was originally aimed for academic genealogy in neuroscience. Social sciences fields, like education, literature, sociology, and economics, are also well represented. Table 13 gives the count of each type of mentorship.

**Table 8:** The top 20 most represented major research areas.

area	researchers	% researchers	researchers matched	% matched
neuroscience	135756	16.7	93769	69.1
chemistry	104450	12.9	85585	81.9
engineering	56898	7.0	45004	79.1
education	56580	7.0	17978	31.8
physics	49582	6.1	37714	76.1
math	35651	4.4	22707	63.7
literature	28257	3.5	7449	26.4
sociology	25453	3.1	12618	49.6
economics	23497	2.9	12841	54.6
computer science	22399	2.8	18315	81.8
cell biology	20970	2.6	18087	86.3
political science	18914	2.3	8654	45.8
theology	17448	2.1	3726	21.4
microbiology	17230	2.1	14759	85.7
phillosopy	17035	2.1	6253	36.7
linguistics	13952	1.7	6685	47.9
nursing	13825	1.7	6207	44.9
phtree	13637	1.7	8986	65.9
anthropology	13471	1.7	6185	45.9
evolution	13417	1.7	10494	78.2

**Table 9:** Performances of genderize.io.

	Validation set	
	F-1	precision
male	0.74	0.59
female	0.77	0.64

**Table 10:** Performances of ethnicity prediction with ethnicolr within race-gender bucket.

	Validation set	
	F-1	precision
Black male	0.03	0.14
Black female	0.03	0.15
Hispanic male	0.72	0.91
Hispanic female	0.55	0.87
White male	0.54	0.37
White female	0.50	0.34
Asian male	0.71	0.96
Asian female	0.62	0.88

**Table 11:** Performances of our gender prediction within race-gender bucket.

	Validation set	
	F-1	precision
Black male	0.98	1.00
Black female	0.97	1.00
Hispanic male	0.96	1.00
Hispanic female	1.00	1.00
White male	0.99	1.00
White female	0.99	1.00
Asian male	0.99	1.00
Asian female	0.97	1.00

**Table 12:** Performances of our race prediction within race-gender bucket.

	Validation set	
	F-1	precision
Black male	0.69	0.64
Black female	0.70	0.63
Hispanic male	0.81	0.94
Hispanic female	0.78	0.90
White male	0.55	0.76
White female	0.55	0.69
Asian male	0.74	0.62
Asian female	0.76	0.66

**Table 13:** Mentorship type definition and statistics.

Mentorship type	Definition	Count
0	Research assistant	18850
1	Graduate student	630439
2	Postdoctoral	68652
3	Research scientist	7402
4	Collaborator	17833

### Validation of linking AFT researchers with MAG authors

Table 8 indicates that we can match the majority of researchers in natural sciences, but for social sciences fields like education, literature, we have lower percentages of researchers matched.

To validate our linking of AFT researchers to MAG authors, we take advantage of the fact that their publications are known to be genuinely authored by them for some AFT researchers. With these publications, we examine if they also appear in the publication list of the corresponding matched MAG author. Here we focus on two subsets of AFT researchers: (1) those with papers verified by AFT website users; and (2) those with ORCID available.

Let us describe the first subset. In our previous works^8,21^, we have automatically linked AFT researchers to publications indexed in PubMed. Those matched papers are then displayed on researchers’ profile pages. AFT website users who have signed into the website can label whether the authorship is correct. We consider these labeled papers as a validation set to test the performance of our AFT-to-MAG matching of authors. To match these papers to MAG, we rely on their DOIs. For papers without DOI but with PMID, we query PubMed to get their DOI^41^.

We can now introduce the measure used to quantify the performance of our matching. Let *a* be an AFT researcher who has at least one verified and *P*_*a*_ the list of her verified papers. Let also *a*′ be the corresponding matched MAG author and *P*_*a*′_ the list of papers found on MAG. We calculate the fraction of *P*_*a*_ that appear in *P*_*a*′_, formally:

1 $${O}_{a}=\frac{\left|{P}_{a}\cap {P}_{a{\prime} }\right|}{\left|{P}_{a}\right|}.$$

**Fig. 4:**
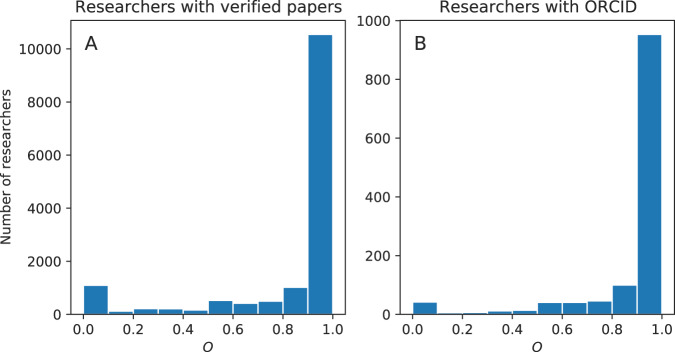
Validation of matching AFT researchers with MAG authors. The measure *O* considers, for an AFT researcher, *a*, the list of papers, *P*_*a*_, genuinely authored by her, and measures the fraction of these papers that also appear in the list of papers of the corresponding matched MAG author. (**A**) Histogram of *O* for 14824 researchers with |P_a | > 0. Here *P*_*a*_ refers to papers that registered AFT website users verify. (**B**) Histogram of *O* for 1262 researchers with ORCID identifiers. *P*_*a*_ represents the list of papers extracted from the orcid.org website. In both cases, we observe that for the vast majority of researchers, most of their papers that they genuinely author can be found in the lists of publications of their matched MAG authors, indicating high accuracy of our matching procedure.

Figure 4A, which plots the histogram of *O*_*a*_ for the first subset of researchers, indicates the validity of our matching process; for the vast majority of researchers, we can find most of their verified papers in the publication lists of their matched MAG authors.

Let us describe the second subset: papers listed on the ORCID website (*P*_*a*_). To get these papers, we download the 2019 ORCID Public Data File (the most recent one)^42^, extract documents authored by researchers, and match extracted papers to MAG using their DOI. Figure 4B shows the histogram of *O*_*a*_ for the second subset of researchers, indicating most of their papers also appear in publication lists of corresponding matched MAG authors.

### Validation of author vector

**Fig. 5:**
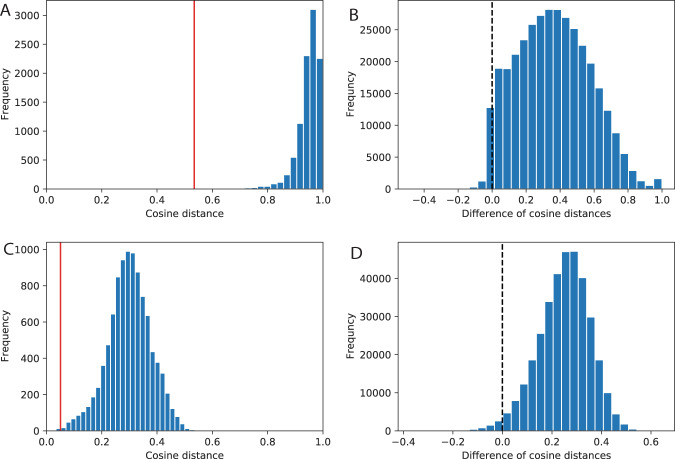
Validation of researcher vectors. (**A**) Histogram of cosine distances of TF-IDF vectors between one researcher *a* and 10 thousand randomly selected researchers. The red vertical line marks the distance between *a* and *a*’s Ph.D. mentor, *b*, indicating that *a* is much closer to her mentor than expected. (**B**) For each PhD mentee, we calculate the difference of *d*(*a*, *c*) and *d*(*a*, *b*), where *d*(*a*,*c*) is the cosine distance between *a* and *c*, a randomly selected researcher. The figure shows the histogram of the differences for all Ph.D. mentees, indicating that they are semantically much closer to their mentors than to random researchers for the vast majority of Ph.D. mentees. (**C**,**D**) The same as A–B, except that researcher vectors are based on the SPECTER algorithm rather than TF-IDF.

We validate researchers’ vectors by comparing distances between researchers who belong to different groups. Specifically, in Fig. 5A, we show that the cosine distance of the TF-IDF vectors of a particular Ph.D. mentee, *a*, and her mentor, *b*, is much smaller than the distances between *a* and randomly selected researchers. Generalizing this systematically, for each Ph.D. mentee, we obtain a triplet *(a*, *b*, *c)* where *c* is a randomly chosen researcher. We then calculate the difference of the distance between *a* and *c*, *d* (*a, c*), and the distance between *a* and *b*, *d*(*a, b*). As we expect, the semantics of a mentee is more similar to her Ph.D. mentor than to a random researcher, and the distance difference is expected to be larger than 0. This pattern is indeed the case for the vast majority (97.4%) of Ph.D. mentees (Fig. 5B). We also replicate these analyses using SPECTER vectors, and the results remain similar (Fig. 5C,D): For 98.4% of Ph.D. mentees, they are semantically closer to their Ph.D. mentors than randomly selected researchers (Fig. 5D). The threshold 0 is located at 1.66 and 2.39 standard deviations away from the mean for the TF-IDF case and SPECTER case, respectively, suggesting that SPECTER is a better representation method.

**Fig. 6:**
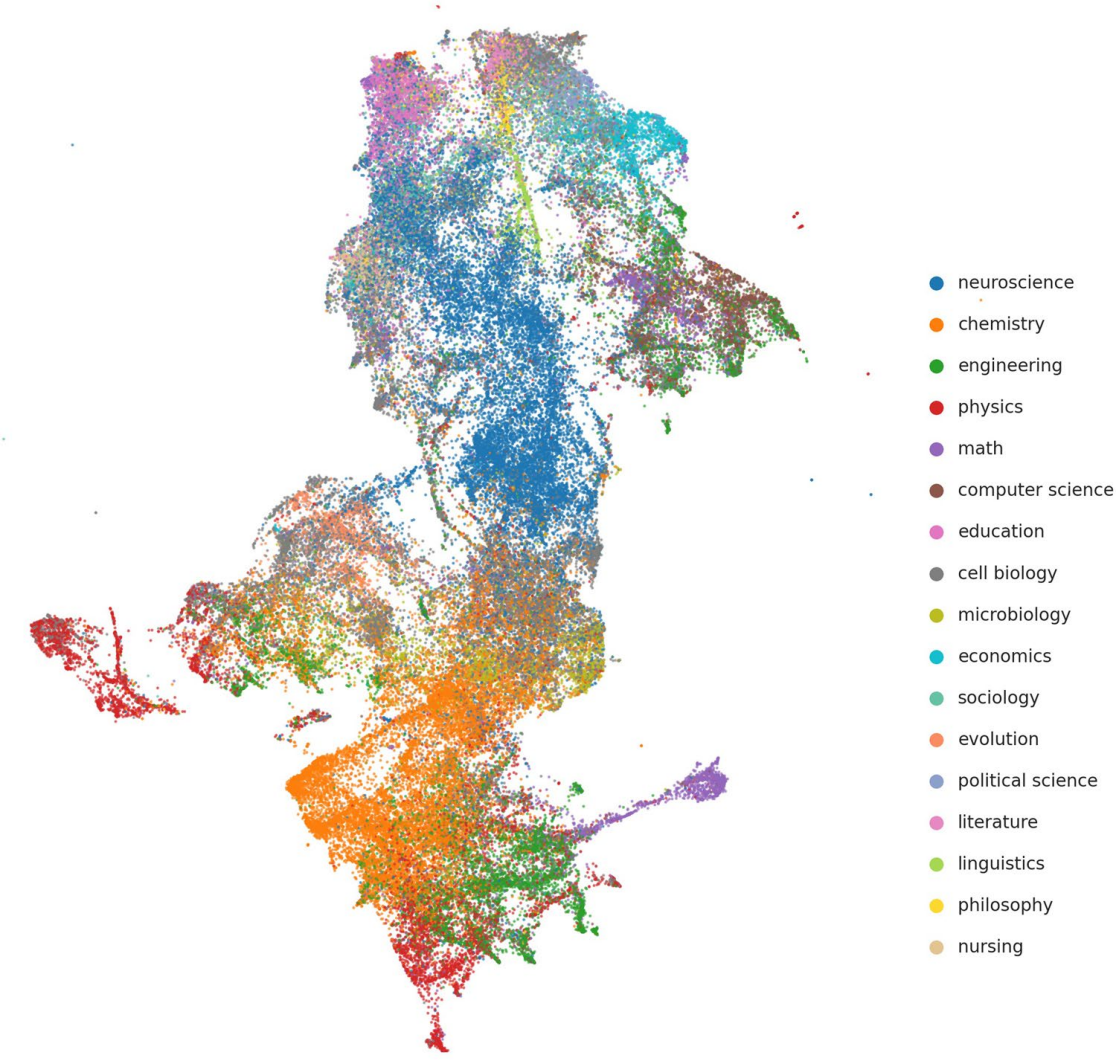
The 2-dimensional projections of researchers’ SPECTER vectors, obtained using UMAP^43^. The figure shows a 20% random sample of all researchers. An interactive version can be found at https://scienceofscience.org/mentorship.

To further show the structure of researchers’ SPECTER vectors, we run the UMAP^43^ dimension reduction technique to obtain 2-dimensional vectors and display them as a scatter plot for a 20% random sample of researchers in Fig. 6. As expected, researchers in the same research area are clustered, meaning that they are semantically closer to each other than researchers from other areas.

## Usage Notes

Users can integrate our data set with MAG to study the role of mentor in mentee’s academic career. MAG provides detailed information about papers and citations, from which users can derive various indicators commonly used in the science of science. We can access MAG data by following the steps outlined on its website^44^. In addition to MAG, other identifiers of publications we provide also facilitate integration with other scholarly databases. In particular, users can use CrossRef API to retrieve metadata of papers using DOI^45^. Also, we can use the E-utilities API provided by the National Library of Medicine to obtain metadata of PubMed articles using PMID^41^.

Users who want to use our released researcher vectors to perform semantic analysis can load the TF-IDF vector file using the SciPy library’s scipy.sparse.load_npz function.

## Data Availability

All the code for generating the dataset and figures is published as IPython notebooks on Github, https://github.com/sciosci/AFT-MAG. All the coding was completed using Python.
